# *Senecio biafrae* defeated Tetracycline-Induced Testicular Toxicity in Adult Male Sprague Dawley Rats

**DOI:** 10.5935/1518-0557.20180054

**Published:** 2018

**Authors:** Sunday Adelakun, Olusegun Omotoso, Julius Aniah, Oyebowale Oyewo

**Affiliations:** 1 Department of Human Anatomy, School of Health and Health Technology, Federal University of Technology, Akure, Nigeria; 2 Department of Anatomy, Ladoke Akintola University of Technology, Ogbomosho, Oyo State, Nigeria; 3 Department of Anatomy, Kogi State University, Anyigba, Kogi State, Nigeria; 4 Department of Anatomy, College of Medicine, University of Abuja, Federal Capital Territory (FCT), Nigeria

**Keywords:** Senecio biafrae, fertility, testis, vitamin C, sperm, rat

## Abstract

**Objective:**

The current study focused on the pro-fertility potential of *Senecio biafrae (Sb)* extract and vitamin C in Male Sprague Dawley (SD) rats with tetracycline-induced infertility.

**Methods:**

A total of 36 male and 36 female adult SD rats were used for this investigation. The male rats randomly assigned to Group A (controls) were given normal saline 2ml/kg. Rats in Groups B, C, D, E, and F were respectively administered [30 mg/kg of body weight (bwt) of tetracycline], [30 mg/kg bwt of tetracycline + 50 mg/kg of vitamin C], [30 mg/kg bwt of tetracycline + 500 mg/kg bwt of *Sb*], [30 mg/kg bwt of tetracycline + 50 mg/kg of vitamin C + 500 mg/kg bwt of *Sb*], and [30 mg/kg bwt of tetracycline reversal] daily for 28 days via gastric gavage. Tested parameters included sperm parameters, hormonal profile, histology, and fertility test.

**Results:**

Significant (*p*<0.05) increases were seen in sperm quality, hormone profile, organ and body weights of the groups treated with vitamin C, *Sb*, and tetracycline. There was derangement in sperm quality, hormone profile, and organ and body weight of the animals in group B. Histoarchtecture of the testes showed normal cellular composition in the germinal epithelium with sperm cells in the lumen and normal interstitium in groups A, C, D, and E. Group F showed abnormal spermatogenesis and poor association of spermatogenic cells, however there was depletion in the seminiferous epithelium in the group treated with tetracycline.

**Conclusion:**

*Senecio biafrae* defeated the deleterious effects of tetracycline on the male reproductive system of rats treated with the drug.

## INTRODUCTION

Medicinal Plants are essential for the development of modern drugs and have been used in daily life to treat diseases all over the world for many years (Ates & Erdogrul, 2003; Müller *et al.,* 2009). Indeed, many of these plants have been used to treat various reproductive ailments such as male and female infertility, a public health concern in Sub-Saharian Africa (Lux, 1976). More than three quarters of the world’s population rely upon complementary and alternative medicine for health care (Edirne *et al.,* 2010). *Senecio biafrae* is one of these plants (Telefo *et al.,* 2011). It is a perennial climbing herb that occurs naturally in African forest zones, from Guinea to Uganda. Its leaves contain various secondary metabolites such as dihydroisocoumarins, terpenoids, sesquiterpenes or amino acids (Tabopda *et al.,* 2009). *Senecio biafrae* is one of the green leafy vegetables consumed in Sierra Leone, Ghana, Benin, Nigeria, Cameroon and Gabon (Adebooye, 2004). Green leafy vegetables are sources of vitamins, minerals, and fiber to local consumers, due to their dietary importance; many scientific studies have been carried out on the potential benefits of these green leaves (Akindahunsi & Salawu, 2005). *Senecio biafrae* is very rich in protein (29%), food fiber, and minerals such as manganese, sodium, potassium, magnesium, and calcium (Dairo & Adanlawo, 2007). It is also known for its therapeutic virtues, notably in Nigeria where it is used in the treatment of diabetes and pulmonary defects (Gbolade, 2009; Iwu, 1993). In the West and Northwest regions of Cameroon, ethnobotanical studies revealed its utilization in the treatment of cases of female infertility (Focho *et al.,* 2009; Tacham, 2000).

Tetracycline is an antibiotic employed clinically in the treatment of bacterial infections. It is known to cause testicular damage, biochemical dysfunction and suspected to induce testicular damage in animals, but there is paucity of data on its effects and mechanism of action on the male reproductive system (Farombi *et al.,* 2008). About 50% of the known causes of primary infertility are attributed to male factors (Kumar & Singh, 2015). However, the etiology of male factor infertility is not easy to define. Environmental pollutants as well as modern day social habits such as smoking, alcohol consumption, and drug abuse have all been associated with male infertility (Sharma *et al.,* 2013).

Infertility refers to the inability to conceive after having regular unprotected sex. Infertility may also refer to the biological inability of an individual to contribute to conception, or to a female who cannot carry a pregnancy to full term. In many countries infertility refers to a couple that has failed to conceive after 12 months of regular sexual intercourse without the use of contraception. Studies indicate that slightly over half of all cases of infertility are a result of female conditions, while the rest are caused by either sperm disorders or unidentified factors (Nordqvist, 2016). To most couples the desire to have their own biological children is strong and compelling. The effects of infertility on these couples can be devastating. Infertility leads to psychological stress, anxiety, and depression (Centers for Disease Control and Prevention [CDC], 2014). Over 186 million couples in developing countries alone (excluding China) are affected by infertility (WHO, 2003). Rates of infertility vary considerably from country to country; in areas more significantly affected, over 25% of the couples may be unable to have children (Okonofua *et al.,* 1997). On a practical level, many families in developing countries depend on their children for economic survival. Therefore, while many people would not consider infertility a disease in itself, it is certainly a social and public health issue as well as an individual problem (WHO, 2003). In Nigeria, data on infertility indicates that disorders in males and females account for an equal proportion of infertility with the male factor being associated with a greater percentage of primary infertility (Hollos *et al.,* 2009). Available evidence revealed that male factor infertility has not been given due prominence in issues of reproductive health (Okonofua *et al.,* 2005; Onyeka *et al.,* 2012).

The present study aims to investigate the possible fertility potential of *Senecio biafrae* extract and vitamin C in male Sprague Dawley rats with tetracycline-induced infertility.

## MATERIALS AND METHODS

Tetracycline tablets (Medrel pharmaceuticals, India) and vitamin C tablets (Emzor Pharmaceuticals, Nigeria) were obtained from the Department of Pharmacy of the State Specialist Hospital, Akure, Ondo State, Nigeria.

### Plant Material

Plant materials were collected from the Research Farm, Faculty of Agricultural Sciences, Ladoke Akintola University of Technology (LAUTECH) Ogbomoso, Oyo State, Nigeria. Samples of *Senecio biafrae* were identified and authenticated by Prof. A.T.J. Ogunkunle of the Department of Pure and Applied Biology and plant voucher specimens were deposited for reference purposes.

### Extraction of plant material

The leaves were thoroughly washed in sterile water and air dried to a constant weight in the laboratory. The air-dried leaves were weighed using a CAMRY (EK5055, India) electronic scale and were milled in an automatic electrical Blender (model FS-323, China) to powdered form. Five hundred grams of milled plant were later soaked in 1000 ml of PBS for 48 hours (Iweala & Okeke, 2005) at room temperature, and the solution was later filtered through cheese cloth and then through Whatman #1 filter paper (Khan *et al*., 2010); the filtrate was concentrated using a rotary evaporator (Rotavapor^®^ R-220) at 42-47ºC.

### Animals

Male and female Sprague Dawley rats procured from the Experimental Animal House, Department of Anatomy, Ladoke Akintola University of Technology, Ogbomoso, Nigeria, were authenticated and used throughout the study. The animals were kept in cages and allowed to acclimatize for a period of two weeks before the commencement of the experiment. The rats were maintained under standard natural photoperiodic condition of twelve hours of darkness and twelve hours of light (D:L; 12:12h dark/light cycle) at room temperature (25-32ºC) and humidity of 50-55% (Yakubu *et al.,* 2008). Their cages were cleaned every day. The rats were fed with standard chow at a recommended dose of 100 g/kg/day as advised by the International Centre of Diarrheal Disease Research, Bangladesh (ICDDR, B). Drinking water was supplied *ad libitum*. The weights of the rats were documented at procurement, during the period of acclimatization, at commencement of administrations, and once a week throughout the period of the experiment, using a CAMRY (EK5055, India) electronic analytical precision scale.

### Experiment design and animal grouping

A total of 36 male and 36 female healthy adult (12-14 weeks old) Sprague Dawley rats weighing 200-220g were used in this study. The male rats were randomly divided into six groups (A, B, C, D, E, and F) with six (n=6) individuals each. The individuals in Group A (controls) were each given normal saline 2ml/kg daily for 28 days. Rats in Groups B, C, D, E, and F were each respectively administered [30 mg/kg of body weight of tetracycline], [30 mg/kg of body weight of tetracycline + 50m g/kg of vitamin C], [30 mg/kg body weight of tetracycline + 500 mg/kg of body weight of *Senecio biafrae*], [30 mg/kg body weight of tetracycline + 50 mg/kg of vitamin C + 500 mg/kg of body weight of *Senecio biafrae*] and [30 mg/kg of body weight of tetracycline reversal] daily for 28 days. The extract was administered once daily for six days within a week via gastric gavage. Reversal group F was left for 28 days after the cessation of treatment with tetracycline to see whether the observed effects were reversible. Female rats were used to copulate with control and treated male rats to test for fertility after administration.

### Animal sacrifice and sample collection

At the time of sacrifice the rats were first weighed and sacrificed by cervical dislocation. The abdominal cavity was opened up through an incision in the abdominal midline to expose the reproductive organs. The testes were excised and trimmed of all fat. Blood samples were collected through cardiac puncture for hormonal assays. The testes and epididymis of the rats were carefully dissected out and weighed independently. The testes from each rat were exposed carefully and removed. They were trimmed free of epididymides and adjoining tissue.

### Semen Analysis

The rats were sacrificed by cervical dislocation. Orchiectomy was performed by open castration. The testes were exposed by incising the tunica vaginalis, and the cauda epididymis was harvested. The cauda epididymis of rats in each of the experimental groups was minced thoroughly in a specimen bottle containing normal saline for a few minutes to allow sperm to become motile and swim out from the cauda epididymis (Saalu *et al.*, 2008).

### Sperm count and Motility studies

Semen was then taken with 1ml pipette and dropped on a clean slide, and covered with cover slips. The slides were examined under a light microscope for sperm motility (Saalu *et al.*, 2008). And with the aid of an improved Neubauer hemocytometer (Deep1/10mm LABART, Germany) counting chamber as described by Pant & Srivastava (2003), the spermatozoa were counted under a light microscope. Counting was done in five Thoma chambers.

### Progressive Assessment

Sperm motility was evaluated across a minimum of five strips of squares within a 10-second observation time per square. Non-motile spermatozoa were first counted and then only sperm that exhibited flagella activity were deemed motile. For thorough assessment of motility, the spermatozoa were classified based on recommendations of the World Health Organization (WHO, 2010) into the following categories: Progressive motility/rapid linear progressive motility (X_0_): Spermatozoa in this category exhibited active movement, either linearly or in a large circle, regardless of speed. Non-progressive motility/slow linear progressive motility (Y_0_): Spermatozoa in this category exhibited flagellar movement but the flagellar force hardly displaced the head of the spermatozoon and consequently the spermatozoon lacked progression or exhibited only minor circular movement.

### Sperm morphology

The method described by Saalu *et al.* (2013) was used to evaluate sperm morphology. Sperm morphology was evaluated with the aid of a light microscope at x400 magnification. Caudal sperm taken from the original dilution for motility were diluted 1:20 with 10% neutral buffered formalin (sigma- Aldrich, Canada). The spermatozoa were categorized in wet preparations using phase contrast optic. In this study a spermatozoon was considered abnormal morphologically if it had a rudimentary tail or a round or detached head; the proportion of morphologically abnormal sperm was expressed as a percentage in relation to morphologically normal sperm.

### Hormone determination

The serum levels of Testosterone (TT), follicule stimulating hormone (FSH) and leutenizing hormone (LH) were measured using commercially available enzyme-linked immunoassay kits (Diagnostic automation Inc, CA) obtained from Randox Laboratories Ltd., Admore Diamond Road, Crumlin, Co., Antrim, United Kingdom, Qt94QY; the kits were used in accordance with manufacturer instructions.

### Testicular histology preparation

The histology of the testes was analyzed by a modification of the method described by Kayode *et al.* (2007). The organs were harvested and fixed in Bouin’s solution for 24 h; then they were transferred to 70% alcohol for dehydration. The tissues were placed in 90% and absolute alcohol and xylene for different times before they were transferred into two changes of molten paraffin wax for 1 hour each in an oven at 65◦C for infiltration. They were subsequently embedded and serial sections cut using a rotary microtome at 5 microns. The tissues were picked up with albumenized slides and allowed to dry on a hot plate for 2 min. The slides were dewaxed with xylene and passed through absolute alcohol (2 changes); 70% alcohol, 50% alcohol, and then water for 5 min. The slides were stained with hematoxylin and eosin. The slides were mounted in DPX. Photomicrographs were taken at a magnification of x100.

### Fertility Test

The fertility test was done using a modification of the method reported by Ligha *et al.* (2012). Each male rat was isolated and paired with a female rat in the first hours of the estrous cycle as determined by vaginal smear examination, and each paired couple was placed in a separate cage. On the following day, the female rats were checked after mating to detect spermatozoa in their vagina by microscopic examination of the vaginal fluid. Females in which a sperm plug was detected the following morning after mating were deemed to be on day one of gestation. The fetuses were removed by ventral laparotomy on the 21^st^ day of gestation and counted.

### Ethical considerations

All experimental procedures followed the recommendations provided in the "Guide for the Care and Use of Laboratory Animals" prepared by the National Academy of Sciences and Published by the National Institute of Health (NIH, 1985).

### Data presentation and statistical analysis

Data were expressed as Mean±SEM. Statistical differences between the groups were evaluated by one-way ANOVA, followed by the Dunnett’s test to compare between treatment and control groups. Differences yielding *p*<0.05 were considered statistically significant. Statistical analyses of data were performed using GraphPad Prism 5 Windows (GraphPad Software, San Diego, California, USA).

## RESULTS

### Changes in body and organ weight

Table 1 shows that the rats treated with tetracycline in Group B (215.9±2.54) did not experience significant increases in body weight in comparison with controls (225.5±3.04); however, the rats in Groups C, D, and E respectively treated with [tetracycline + vitamin C], [tetracycline + *Senecio biafrae*], and [tetracycline + vitamin C + *Senecio biafrae*] saw significant increases in body weight when compared with controls (*p*<0.05). The body weight of the rats in Group F (tetracycline reversal) also increased but not significantly when compared with controls. Testis, epididymis and seminal vesicle weight significantly decreased in Group B when compared with controls (*p*<0.05); Groups C, D, E, and F showed no significant difference in comparison with controls, but had significant increases in comparison with Group B.

**Table 1:** Effect of aqueous extract of *Senecio biafrae* leaves on body and organ weight of adult male Sprague Dawley rats with tetracycline-induced infertility after 28 days of administration.

Parameters	Groups					
	A	B	C	D	E	F
Initial body weight (g)	213.8±3.04	209.8±1.38	207.0±1.53	208.0±1.24	214.3±4.19	211.3±3.05
Final body weight (g)	225.5±3.09	215.9±2.54	249.9±6.65^α^	277.1±7.91^α^	256.2±7.22^α^	222.6±3.01
Body weight difference (g)	11.7± 0.05	6.1±1.26	42.9±5.12^α^	69.1±6.67^α^	41.9±3.03^α^	11.3±0.04
Testis	0.84±0.02	0.68±0.04^β^	0.81±0.02	0.74±0.02	0.80±0.02**	1.90±0.02**
Epididymis	0.27±0.08	0.05±0.00^β^	0.12±0.05	0.33±0.05	0.36±0.06**	0.26±0.08
Seminal Vesicle	0.40±0.02	0.16±0.05^β^	0.40±0.04	0.36±0.02	0.37±0.02**	0.33±0.05**

Values are expressed as Mean ± S.E.M, n=6 in each group

α significantly greater than control group at *p*<0.05

β significantly lower than control group

** significantly dissimilar from group B One-Way ANOVA.

The organo-somatic index (OSI) was expressed as a percentage of the total body weight in relation to the weight of the target organs, OSI = (Organ weight/total body weight) × 100. A (Control): 2ml/kg of body weight of normal saline B: 30 mg/kg of body weight of Tetracycline C: 30 mg/kg of body weight of Tetracycline and 50 mg/kg of body weight of vitamin C D: 30 mg/kg of body weight of Tetracycline and 500 mg/kg of body weight of *Senecio biafrae* extract E: 30 mg/kg of body weight of Tetracycline + 50 mg/kg of body weight of vitamin C + 500 mg/kg of body weight of *Senecio biafrae* extract F: 30 mg/kg of body weight of Tetracycline reversal

### Sperm count and sperm motility

The mean values for sperm count and sperm motility in controls given 2ml/kg of normal saline orally per day were 79.65±2.50 and 67.09±3.80, respectively. Group B - given 30 mg/kg of body weight of tetracycline - had significant decreases in mean sperm count and sperm motility (*p*<0.05) when compared with controls. Significant increases (*p*<0.05) in mean sperm count and motility (67.38±3.59, 68.95±3.40), (87.19±2.03, 83.72±2.45) and (89.45±4.1, 90.12±3.12) were seen in Groups C, D, and E, respectively treated with [30 mg/kg of body weight of tetracycline + 50mg/kg of body weight vitamin C], [30 mg/kg of body weight of tetracycline + 500 mg/kg of body weight of *Senecio biafrae*], and [30 mg/kg of body weight of tetracycline + 50mg/kg of body weight vitamin C + 500mg/kg of body weight of *Senecio biafrae*] when compared with controls. The mean values for Group F increased but not significantly in comparison with controls. When compared to Group B, the mean values seen in Group F increased significantly. The mean sperm count and motility values for Groups D and E - (87.19±2.03, 83.72±2.45) and (89.45±4.1,90.12±3.12) - were significantly greater than the mean values found for Group C - (67.38±3.59 and 68.95±3.40) (*p*<0.05) (Table 2).

**Table 2:** Effect of aqueous extract of Senecio biafrae leaves on the sperm profile of adult male Sprague Dawley rats with tetracycline-induced infertility after 28 days of administration.

Parameters	Groups					
	A	B	C	D	E	F
Sperm court (x 10^6^ /ml)	79.65±2.50	33.03±2.34^β^	67.38±3.59^β^	87.19±2.03^¥^	89.45±4.15^α^	72.35 ± 2.32^¥^
Sperm motility (%)	67.09±3.80	34.45±2.36^β^	68.95±3.40	83.72±2.45^α,^^¥^	90.12± 3.12^α^	63.11 ±3.21^¥^
Progressivity	X_0_	Y_0_	X_0_	X_0_	X_0_	X_0_
Normal morphology(%)	74.86±1.98	32.34±2.43^β^	75.11±3.10	85.68±2.06^¥^	87.34±6.23^α,¥^	68.45± 5.13^¥^
Abnormal morphology(%)	22.56±2.07	68.50±2.01^α^	22.31±2.44	14.61±1.39^β,^^¥^	12.57±2.32^β,¥^	25.43±3.12^¥^
Sperm live /dead ratio (%)	76.45±2.03	28.75±1.28^β^	77.46±3.71	87.47±2.42^¥^	89.35±4.12^α,¥^	69.21±5.23^α,¥^

Values are expressed as Mean ± S.E.M, n=6 in each group

α significantly greater than control group

β significantly lower than control group

¥ significantly different from group C at *p*<0.05. One-Way ANOVA.

X_0_: Rapid linear progressive motility Y_0_: Slow linear progressive motility A: (Control) 2ml/kg of body weight of normal saline B: 30 mg/kg of body weight of Tetracycline C: 30 mg/kg of body weight of Tetracycline and 50 mg/kg of body weight of vitamin C D: 30 mg/kg of body weight of Tetracycline and 500 mg/kg of body weight of *Senecio biafrae* extract E: 30mg/kg of body weight of Tetracycline + 50 mg/kg of body weight of vitamin C + 500 mg/kg body weight of *Senecio biafrae* extract F: 30 mg/kg of body weight of Tetracycline reversal

### Sperm progressivity and sperm morphology

There were significant (*p*<0.05) differences in sperm progressivity across the groups. The proportion of normal sperm significantly increased in Groups C, D, and E when compared with controls. A significant decrease in the proportion of abnormal sperm in Groups D and E - (14.61±1.39) and (12.57±2.32) - was seen in relation to Groups A, B, C, and F (22.56±2.07, 68.50±2.01, 22.31±2.44 and 25.43±3.12); however, there was a significant increase in the proportion of abnormal sperm in Group B (68.50±2.01) treated with 30mg/kg of tetracycline when compared with controls in Group A (22.56±2.07) (*p*<0.05) (Table 2).

### Sperm live/dead ratio

Sperm live/dead ratio increased significantly in Groups C, D, E, and F when compared with controls; the sperm live/dead ratio significantly decreased in Group B treated with 30mg/kg of body weight of tetracycline in comparison with controls (*p*<0.05) (Table 2).

### Serum testosterone, follicle stimulating hormone and luteinizing hormone levels

Table 3 shows that controls in Group A treated with 2 ml/kg of normal saline had a mean testosterone level of 1.94±0.14. No significant increases were seen in the mean testosterone levels of Groups C, D, E, and F (1.67±0.11, 1.81±0.08, 1.92±0.06 and 1.81±0.12) respectively when compared to controls. However, a significant decrease was detected in the mean testosterone level of the subjects in Group B treated with 30 mg/kg of body weight of tetracycline when compared with controls. In addition, the mean testosterone level seen in Groups C, D, E, and F were significantly increased when compared with Group B treated with tetracycline. The mean serum follicle stimulating hormone (FSH) level seen in Group B (0.15±0.01) was significantly lower than the mean level seen in controls (*p*<0.05). However, a significant increase in mean FSH level was seen in Groups C, D, E, and F in relation to Group B. In the same vein, the mean luteinizing hormone (LH) level of Group B treated with 30 mg/kg of body weight of tetracycline significantly decreased when compared to controls in Group A (*p*<0.05); however, the mean LH levels of Group C, D, E and F (0.19±0.01, 0.21±0.02, 0.25±0.04 and 0.16±0.02) were significantly increased when compared with Group B (0.11±0.01) (*p*<0.05).

**Table 3:** Effect of aqueous extract of *Senecio biafrae* leaves on the hormone profile of adult male Sprague Dawley rats with tetracycline-induced infertility after 28 days of administration

Parameters	Groups					
	A	B	C	D	E	F
TT (ng/ml)	1.94±0.14	0.83±0.14^β^	1.67±0.11**	1.81±0.08**	1.92±0.06**	1.81±0.12**
FSH (mlU)	0.24±0.02	0.15±0.01^β^	0.24±0.02**	0.29±0.02**	0.30± 0.01**	0.21±0.02**
LH (mlU)	0.18±0.01	0.11±0.01^β^	0.19±0.01**	0.21±0.02**	0.25±0.04**	0.16±0.02**

Values are expressed as Mean ± S.E.M, n=6 in each group

** significantly dissimilar from group B

β significantly lower than control group, at P<0.05. One-Way ANOVA.

TT: testosterone, FSH: follicle stimulating hormone, LH: leutenizing hormone, A: (Control) 2 ml/kg of body weight of normal saline B: 30 mg/kg of body weight of Tetracycline C: 30 mg/kg of body weight of Tetracycline and 50 mg/kg of body weight of vitamin C D: 30 mg/kg of body weight of Tetracycline and 500 mg/kg of body weight of *Senecio biafrae* extract E: 30 mg/kg of body weight of Tetracycline + 50mg/kg of body weight of vitamin C + 500 mg/kg body weight of *Senecio biafrae* extract F: 30mg/kg of body weight of Tetracycline reversal

### Fertility test in control and treated rats

**Figure 1:**
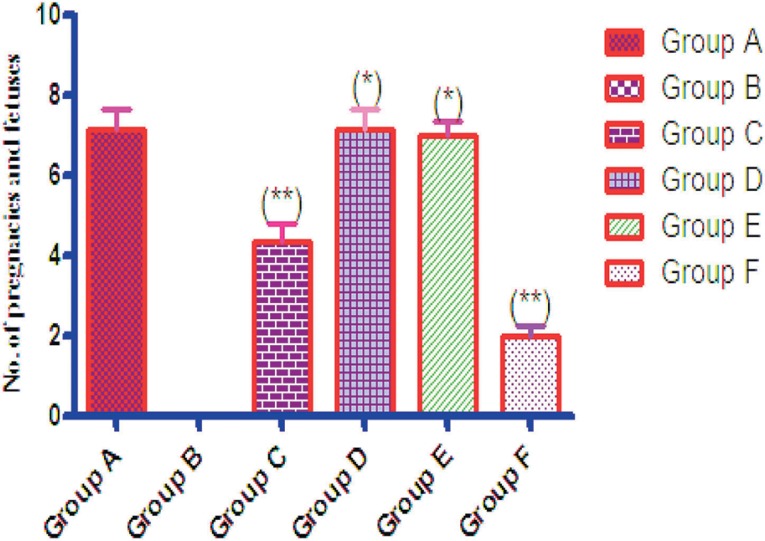
Fertility data of control and treated groups. Values are expressed as Mean ± S.E.M, n=6 in each group (**): significantly dissimilar from control group (*) significantly dissimilar from control group B at *p*< 0.05. One-Way ANOVA. A: (Control) 2ml/kg of body weight of normal saline B: 30 mg/kg of body weight of Tetracycline C: 30mg/kg of body weight of Tetracycline and 50 mg/kg of body weight of vitamin C D: 30mg/kg of body weight of Tetracycline and 500 mg/kg of body weight of *Senecio biafrae* extract E: 30mg/kg of body weight of Tetracycline + 50 mg/kg of body weight of vitamin C +500 mg/kg of body weight of *Senecio biafrae* extract F: 30mg/kg of body weight of Tetracycline reversal.

The rats in Group B treated with 30 mg/kg of body weight of tetracycline had impaired fertility, since over 90% of the female rats with confirmed copulation were unable to get pregnant. The rats in Group D given 30 mg/kg of body weight of tetracycline and 500 mg/kg of body weight of *Senecio biafrae* did not suffer with impaired fertility, since all the female rats got pregnant and produced at least six fetuses each. There was a significant decrease in the number of fetuses produced in Group C treated with 50 mg/kg of body weight of vitamin C and 30 mg/kg of body weight of tetracycline (*p*<0.05) when compared with controls. The experimental group treated with 30 mg/kg of body weight of tetracycline + 50 mg/body weight of vitamin C + 500 mg/kg of body weight of *Senecio biafrae* produced more fetuses than the rats in Groups B and F. The number of pregnancies and fetuses was significantly lower in Group F (tetracycline reversal group) than in Groups C, D, E, and among controls (*p*<0.05) (Figure 1).

### Testicular histology

**Figure 2:**
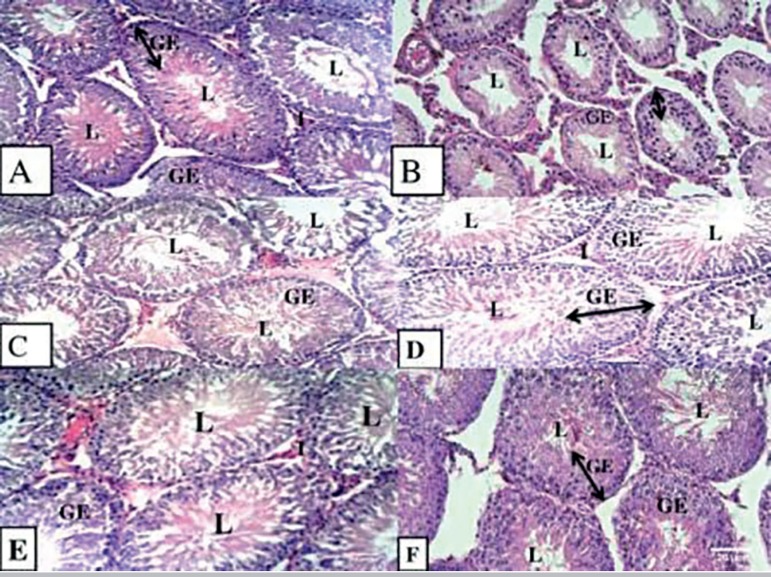
Photomicrographs of testis (×100) after 28 days of administration. Normal cellularity in germinal epithelium (GE), lumen (L) filled with sperm cells and interstitial cells of Leydig in the interstitium (I) in groups A, C (30 mg/kg of body weight of Tetracycline + 50 mg/kg of body weight of vitamin C), D (30 mg/kg of body weight of Tetracycline + 500 mg/kg of body weight of *Senecio biafrae* extract), and E (30mg/kg of body weight of Tetracycline + 50mg/kg of body weight of vitamin C + 500 mg/kg body weight of *Senecio biafrae* extract)]. Group F (30mg/kg of body weight of Tetracycline reversal) had abnormal spermatogenesis, poor association and low density of spermatogenic cells Group B (30 mg/kg of body weight of Tetracycline) showing depleted spermatogenic cells of the germinal epithelium (GE).

Cross sections of the testes of the rats after 28 days of treatment showed that Groups A, C, D, and E had normal germinal epithelium cellular composition with sperm cells in the lumen and normal interstitium. Group F (30 mg/kg of body weight of tetracycline reversal) had abnormal spermatogenesis, poor association, and low density of spermatogenic cells. However, the subjects in Group B (30 mg/kg of body weight of tetracycline alone) had a marked depletion of spermatogenic cells (Figure 2).

## DISCUSSION

Some African populations use *Senecio biafrae* on account of the plant’s nutritional and pharmacological properties and phytochemical constituents (Burkill, 1985; Dairo & Adanlawo, 2007). In this study, administration of *Senecio biafrae* significantly increased the body weight of treated rats compared with controls. This finding is in agreement with the report of Shenoy & Goyal (2002) that *Senecio biafrae* extract possesses may be used to manage glucose levels and control muscle wasting and induced adipogenesis. The body weight of the rats treated with tetracycline was not significantly greater than controls; the mean body weight of the tetracycline reversal group also increased but not significantly when compared with controls, but oral tetracycline significant decreased the weight of the testes, epididymis, and seminal vesicles of treated rats when compared with controls, as also reported by Ajibade *et al.* (2011) and Farombi *et al.* (2008). The decrease in the weight of testes, epididymis, and seminal vesicles was due to decreased cellular activity in the testes. According to Shittu *et al*. (2007), decreased or increased cellular activity is a key factor in the evaluation of organ weight.

In our study, administration of tetracycline (*p*<0.05) significantly reduced sperm count, sperm motility, percent normal morphology, and percent live sperm. Our findings were in agreement with previous reports on the adverse effects of antibiotics on male reproductive function (Hargreaves *et al.,* 1998; Schlegel *et al.,* 1991; Timmermans, 1974). In the same vein, administration of metronidazole and tetracycline significantly decreased the weight of the epididymis, sperm count, motility, and serum testosterone levels (Raji *et al.,* 2007). Significant reduction of sperm count and sperm motility after administration of tetracycline subjected the spermatozoa to increased damage induced by oxidative stress, because their plasma membranes contain large quantities of polyunsaturated fatty acids (PUFAs) (Alvarez & Storey, 1995; Bansal & Bilaspuri, 2011) and their cytoplasm contains low concentrations of scavenging enzymes (Saleh & Agarwal, 2002; Sharma & Agarwal, 1996). Increased formation of reactive oxygen species (ROS) has been correlated with reduced sperm motility (Aitken *et al.,* 1989; Armstrong *et al*., 1999). ROS and reduced motility might be linked through a cascade of events that results in rapid loss of intracellular ATP leading to axonemal damage and sperm immobilization (Bansal & Bilaspuri, 2011; de Lamirande & Gagnon, 1995). However, our study showed improved sperm count, motility, percent normal morphology, and percent live sperm in the Group given *Senecio biafrae* combined with vitamin C and in the reversal group when compared with controls.

The finding that the herbal antioxidants in *Senecio biafrae* increased sperm quality parameters such as population, morphology, and motility in rats with tetracycline-induced infertility was in agreement with the findings reported by Henkel (2005) and Khaki *et al.* (2010). These authors reported that herbal antioxidants eliminated and suppressed ROS formation. Reduction of ROS is a crucial factor in the production of sperm cells and in fertility optimization. Therefore, the administration of *Senecio biafrae* might increase glucose metabolism and support the production of pyruvate, a compound known as the preferred substrate for sperm cell activity and survival. We therefore deduced from our findings that administration of *Senecio biafrae* extract combined with vitamin C increased spermatogenesis in rats with tetracycline-induced infertility, yielding normal reproductive function. This result indicated that *Senecio biafrae* extract and vitamin C have an effect on the mitochondria found in the body of the spermatozoon where energy is synthesized in the form of adenosine triphosphate to increase sperm motility (Duke, 1997). One might hypothesize that the effect of *Senecio biafrae* combined with ascorbic acid on spermatogenesis seen in this study was due to the fact that such agent allegedly works through the hypothalamus-pituitary-gonadal axis. Several studies have reported a protective effect of di etary antioxidants and vitamins A, B, C, and E on sperm DNA against free radicals and improvement of the blood‒testis barrier stability. Since *Senecio biafrae* elevates serum secretion of FSH, LH and testosterone in infertile rats, it might enhance fertility their parameters (Jedlinska-Krakowska *et al.,* 2006; Khaki *et al.,* 2010). The observed increase might be ascribed to the importance of *Senecio biafrae* as a potent antioxidant and free radical scavenger. Increased serum hormone level suggests the existence of a modulating effect of *Senecio biafrae* extract in rats. It has been shown that treatment with antioxidants improves steroidogenesis by enhancing the primary effect of the endocrine function of Leydig cells along with increased circulatory testosterone and stimulation of spermatogenesis (Saalu *et al*., 2013). Reductions in testosterone, FSH, and LH by tetracycline might result from tetracycline reaching the blood-testis barrier and gaining access to the germ cells in the seminiferous tubules, as previously described in the literature (Dixon & Lee, 1973). The blood-testis barrier was possibly an important aspect when considering reproductive and mutagenic effects of drugs and envinronmental chemicals. The permeablity characteristics of the blood-testis barrier are generally similar the traits regulating membrane permeability in the central nervous system (Okumura *et al.,* 1975).

In our study, tetracycline depleted spermatogenic cells and reduced the volume density of the germinal epithelium. This is in concert with the previous study by Popoola *et al.* (2014), in which the administration of tetracycline decreased the number of Leydig cells in the testicles, thus possibly decreasing the testosterone level of the rats included in the study. Spermatogenesis is dramatically depreciated as the Leydig cells that help with testosterone production are affected. We therefore deduced that tetracycline inhibited the proliferative activity of the spermatogonia in all stages of the cycle in the seminiferous tubules, degenerating germ cells and decreasing the number of Leydig cells. It has been reported that testosterone produced by the interstitial cells of Leydig is a necessary prerequisite for the maintenance of established spermatogenesis (Zirkin, 1998). It has been observed that decreased cellularity in the interstitium of the testes of rats treated with tetracycline alone might lead to decreases in testosterone and, consequently, poor spermatogenesis. However, rats given aqueous extract of *Senecio biafrae* leaves maintained the histoarchitecture of their testes, increased the proliferative activity of spermatogonia, and showed better association and higher density of spermatogenic cells when compared with controls. From our observation, aqueous leaf extract of *Senecio biafrae* administered concomitantly with vitamin C and tetracycline protected the reproductive organs against the harmful effects of tetracycline. The protective nature of *Senecio biafrae* is enhanced by some of its phytochemical constituents in the presence of ascorbic acid, known for its protective effect on cell membranes and scavenging effects on free radicals (Eskenazi *et al.,* 2005).

Furthermore, the male rats treated with tetracycline for the period of the study suffered significantly with impaired reproductive system development and maturation, and were unable to impregnate female rats after mating. However, the improvement in fertility in the groups administered tetracycline, vitamin C, and *Senecio biafrae* shows that vitamin C and *Senecio biafrae* contain powerful antioxidants that protect against the oxidative stress induced by tetracycline. We therefore deduced from our findings that *Senecio biafrae* improved sperm and hormone profiles. The administration of vitamin C to rats reportedly improves sperm profiles (Sanghishetti *et al.,* 2014), as supported by our findings.

The findings observed in our study showed that tetracycline produced adverse effects on the testes of Sprague Dawley rats, suggesting that protocols with high doses of tetracycline might result in male infertility. Previous reports on tetracycline concur with the findings of this study. Tetracycline is therefore toxic to the testes and caution should be exercised when administration is required during reproductive age, as tetracycline tends to increase the risk of infertility. This study therefore recommends against the prescription of tetracycline for long periods of time to males. Men taking the drug should be properly educated on the negative effects it might have on the male reproductive system. However, since *Senecio biafrae* has antioxidant components that alter the damage done by tetracycline, male users of the drug can rely on this naturally available plant as a supplement during treatment. Therefore, we can deduce from our findings that *Senecio biafrae* tentatively mitigates the effects of tetracycline on the testes of rats. This study thus confirms the positive effects of *Senecio biafrae* on infertility and sperm quality parameters.
